# Dose Optimization of Teicoplanin for Critically Ill Patients With Renal Dysfunction and Continuous Renal Replacement Therapy: Experience From a Prospective Interventional Study

**DOI:** 10.3389/fphar.2022.817401

**Published:** 2022-03-08

**Authors:** Lu Shi, Zhiwei Zhuang, Lufen Duan, Chenqi Zhu, Hongzhi Xue, Xiao Wang, Xiaowen Xu, Yunlong Yuan, Ling Shi, Jiahui Li, Jiantong Sun, Xin Liu, Qin Zhou, Jian Lu, Lian Tang

**Affiliations:** ^1^ Department of Pharmacy, the Affiliated Suzhou Hospital of Nanjing Medical University, Suzhou Municipal Hospital, Suzhou, China; ^2^ Emergent Intensive Care Unit, the Affiliated Suzhou Hospital of Nanjing Medical University, Suzhou Municipal Hospital, Suzhou, China; ^3^ Intensive Care Unit, the Affiliated Suzhou Hospital of Nanjing Medical University, Suzhou Municipal Hospital, Suzhou, China; ^4^ Medical Laboratory, the Affiliated Suzhou Hospital of Nanjing Medical University, Suzhou Municipal Hospital, Suzhou, China

**Keywords:** teicoplanin, renal dysfunction, continuous renal replacement therapy, therapeutic drug monitoring, high loading dose, albumin, filtration coefficient

## Abstract

**Background:** Due to the lack of updated information on teicoplanin (TEI) for continuous renal replacement therapy (CRRT), no exact dosage regimen has been recommended. The aim of this study was to optimize the dosage regimen of TEI in renal dysfunction patients with or without CRRT, evaluate the influence factors of the eradication of Gram-positive bacteria, and evaluate the effect of CRRT on the clearance of TEI.

**Methods:** Patients with renal dysfunction receiving TEI treatment in the ICU were prospectively recruited and divided into CRRT and non-CRRT groups. Logistic regression analysis was used to screen the factors affecting the eradication of Gram-positive bacteria. The filtrate concentration of the CRRT group was measured at the time of TEI C_min_, and the filtration coefficient of TEI was calculated to evaluate the effect of CRRT on the clearance of TEI.

**Results:** A total of 106 patients were included, 40 cases in the CRRT group and 66 cases in the non-CRRT group. After giving high-loading doses of TEI, 75.8 and 70% of TEI C_min_ in the non-CRRT and CRRT groups reached the range of 10–30 mg/L before the 3rd dose, respectively. The risk of G^+^ bacteria being uneradicated was higher while the APACHEⅡscore was higher than 22.5. The albumin level before the start of TEI administration and before the 6th–8th dose was lower than 32.8 g/L and 29.3 g/L, respectively, and C_min_ before the 3rd dose and 6th–8th dose was lower than 13.2 mg/L and 17.1 mg/L, respectively, with the duration of TEI therapy shorter than 10.5 days. The correlation coefficient (r) was 0.6490 between C_min_ before the 3rd dose and the albumin level (*p* < 0.001). The filtration coefficient of TEI was 10.7 ± 2.4% at C_min_ and 11.1 ± 2.5% at C_max_. The GFR had no correlation with the filtration coefficient (r = −0.06204; r = −0.08059). The clearance of TEI in CRRT patients was negatively correlated with the albumin level (r = −0.6305, *p* = 0.0013).

**Conclusion:** The early stage of the albumin level can significantly affect the initial C_min_ and clinical efficacy of TEI, and also had effect on the clearance of TEI by CRRT. The filtration coefficient of TEI was stable, even with a higher ultrafiltration rate.

## Introduction

Severe acute kidney injury (AKI), especially caused or accompanied by sepsis, is associated with prolonged hospitalization, progression to chronic kidney disease (CKD), financial burden, and a high mortality rate. Many severe AKI patients need to be treated with continuous renal replacement therapy (CRRT) and antimicrobials simultaneously (Karkar and Ronco, 2020). Optimizing antimicrobial therapy in CRRT patients can be challenging because of unpredictable pathophysiological changes, different CRRT approaches, and residual renal functions which can alter the pharmacokinetics (PK) and pharmacodynamics (PD) of antimicrobials (Hoff et al., 2020). Data on drug clearance to guide antimicrobial dosing in CRRT patients are limited. To optimize antibiotic exposure and maximize effectiveness, it is important to individualize the antimicrobial regimen given to the patient and the method of CRRT utilization.

Teicoplanin (TEI) is a glycopeptide antibiotic which is effective against Gram-positive bacteria. Compared with vancomycin, TEI has similar clinical efficacy with advantages in renal toxicity and skin rashes (Kato-Hayashi et al., 2019; Kaur et al., 2021). It has a high protein binding rate of about 90% and long serum elimination half-life of about 50 h requiring the loading dose to quickly reach a stationary state serum concentration (Nakamura et al., 2015; Tang et al., 2020). Our previous studies indicated that the trough concentration (C_min_) of TEI is correlated with the clinical efficacy of TEI therapy (Tang et al., 2020; Ren et al., 2021). It has been recommended that the C_min_ of TEI (at least 10 μg/ml is required for non-complicated MRSA infections) should be maintained at 15–30 μg/ml for most of the methicillin-resistant *Staphylococcus aureus* (MRSA) infections (Abdul-Aziz et al., 2020; Hanai et al., 2021). Our previous studies suggested that a high-loading and maintenance dosage regimen (6–12 mg/kg, q12 h × 3 doses and 6–12 mg/kg qd) is required to maintain the C_min_ of TEI in its therapeutic range at the early stage of treatment, and no reduction in the loading dose is required for renal dysfunction patients (Tang et al., 2020; Ren et al., 2021). The maintenance dose is stratified by renal function. In addition to C_min_, studies about whether other factors affected the clinical efficacy of TEI treatment were limited.

TEI is a medium molecular agent mainly eliminated by the kidney and cannot be eliminated by hemodialysis (Papaioannou et al., 2002). However, the clearance of TEI had individual differences in the ultrafiltration rate (UFR), mode, and even in the filtrating function of CRRT; thus, the recommended dosage is controversial (Bellmann et al., 2010; Lim et al., 2019; Li et al., 2020; Oda et al., 2020). Considering the large molecular weight of TEI, CVVHD may not eliminate TEI efficiently. An early study indicated a loading dose of 800 mg and a maintenance dose of 400 mg q48-72 h under CVVHD (Li et al., 2020). CVVHDF could partly eliminate TEI according to the pharmacokinetic study; however, the TEI concentration varied greatly among CVVHDF patients (Bellmann et al., 2010; Lim et al., 2019; Oda et al., 2020). Current recommendations of patients receiving CRRT originate from the pharmacokinetic estimate of studies with a few patients and different modes of CRRT that are used today (Bellmann et al., 2010; Lim et al., 2019; Li et al., 2020; Oda et al., 2020). Insufficient current pharmacokinetic data limit evidence-based dose recommendations for impaired renal function (IRF) and CRRT patients. Due to a lack of updated information on TEI during CRRT on the CVVH mode, no exact dosage regimen was recommended for CRRT. Therefore, the objective of this prospective interventional study was to optimize the dosage regimen of TEI in renal dysfunction patients receiving CRRT (CVVH mode) or patients not receiving CRRT, and evaluate the factors influencing the eradication of Gram-positive bacteria. The PK parameters and filtration coefficient of TEI were also assessed in CRRT patients.

## Materials and Methods

The protocol for this prospective study was approved by the Ethics Committee of the Affiliated Suzhou Hospital of Nanjing Medical University (Approval No. IEC-C-008-A07-V1.0) and registered in the Chinese Clinical Trial Registration Center (registration number: ChiCTR-2000038259) prior to enrolling the patients into the intervention group. Information about the TEI treatments, therapeutic drug monitoring (TDM), and laboratory indicators was collected after written informed consent of the patients or their families was obtained.

### Study Population

The inpatients with critically ill infections who received TEI therapy in the ICU of the Affiliated Suzhou Hospital of Nanjing Medical University from January 2019 to August 2021 were prospectively recruited. The following are the inclusion criteria: 1) the cultured pathogen of the infected site must be multiple-resistant Gram-positive bacteria, 2) patients should have been treated with TEI for anti-infection therapy, 3) renal dysfunction (Kidney Disease: Improving Global Outcomes (KDIGO) CKD Work Group, 2013) [creatinine clearance rate (CrCl) ≤ 60 ml/min/1.73 m^2^, according to the CKD-EPI equation] or CRRT for AKI, and 4) age >18 years. The following are the exclusion criteria: 1) urine volume >0.5 ml/kg/h in patients during CRRT, 2) patients receiving extracorporeal membrane oxygenation during CRRT, 3) patients who underwent CRRT for non-acute kidney injury, 4) patients with chronic renal failure and underwent regular hemodialysis, 5) CRRT converted to intermittent RRT before TEI treatment, 6) TEI treatment less than three days, or 7) the mode of CRRT was other than CVVH. Patients were divided into the following two groups according to whether they received CRRT: CRRT and non-CRRT groups.

In our study, we calculated CrCl according to the CKD-EPI equation, taking into account Scr, gender, and age, as follows, where Scr is the level of serum creatinine from the first day of TEI therapy:

Female: Serum creatinine µmol/l (mg/dl) ≤ 62 (≤ 0.7):

$$\text{CrCl}=144\times {\left(\frac{Scr}{0.7}\right)}^{-0.329}\times 0.993^{age},$$ (1)

Female: Serum creatinine µmol/l (mg/dl) > 62 (> 0.7):

$$\text{CrCl}=144\times {\left(\frac{Scr}{0.7}\right)}^{-1.209}\times 0.993^{age},$$ (2)

Male: Serum creatinine µmol/l (mg/dl) ≤ 80 (≤ 0.9):

$$\text{CrCl}=141\times {\left(\frac{Scr}{0.9}\right)}^{-0.411}\times 0.993^{age},$$ (3)

Male: Serum creatinine µmol/l (mg/dl) > 80 (> 0.9):

$$\text{CrCl}=141\times {\left(\frac{Scr}{0.9}\right)}^{-1.209}\times 0.993^{age},$$ (4)

Scr(umol/L)=72×Scr(mg/dL), (5)

### Drug Administration

The dosage regimens of TEI in enrolled patients were as follows: 1) loading dose: each patient was given 8–10 mg/kg q12 h × 3 doses; 2) initial maintenance dose: (a) in the non-CRRT group, 6–8 mg/kg qd was given for patients with CrCl at 30–60 ml/min/1.73 m^2^, qd; 6–8 mg/kg qd for patients with CrCl at 10–29 ml/min/1.73 m^2^, and (b) in the CRRT group, 6–8 mg/kg, qd; 3) adjusted maintenance dose: clinical pharmacists advised physicians to adjust the dosage regimen when the initial C_min_ was out of the target range. The maintenance dose was adjusted individually according to TDM and clinical efficacy evaluation of TEI.

### Determination Methods of Plasma Concentration of TEI

The steady-state plasma C_min_ of TEI was determined before the 3rd dose of the loading dose. The sampling time was half an hour before the administration. Again, the C_min_ needs to be determined 72 h after adjusting the administration regimen or the 6th–8th dose of the maintenance dosage regimen. The target C_min_ range of TEI is 10–30 mg/L.

We took a 4-ml venous blood sample in a tube containing the EDTA anticoagulant and centrifuged it immediately at 12,000 r/min for 10 min. The supernatant was taken out and stored at −80°C. The plasma concentration of TEI was determined by using HPLC. The chromatographic separation was performed by using Waters Symmetry C18 column (250 × 4.6 mm, 5 µm) (Waters, USA). Mobile phase was 0.01 mol/L NaH_2_PO_4_ (pH = 3.3) and acetonitrile (75: 25) under a flow rate of 1.0 ml/min. The detection wavelength was 215 nm or 240 nm, the column compartment was kept at 35°C, and the injection volume was 0.020 ml.

### Recording Indicators

Patients’ data were obtained from the inpatients’ medical database. The main analysis indicators of each enrolled patient were recorded as follows: 1) demographic data: gender, age, weight, the APACHE Ⅱ score at the start of TEI treatment, and lactic acid in the blood gas during TEI treatment; 2) infection diagnosis, other clinical diagnoses, and the infection site; 3) the initial and reviewed pathogenic culture, drug susceptibility, and imaging examination; 4) the treatment of TEI, including the beginning time, loading dosage, and initial and adjusted maintenance dose regimens; 5) steady-state plasma C_min_ and ultrafiltrate concentrations after the loading dose and initial or adjusted regimens and the PK parameters of TEI in CRRT patients; 6) changes in biochemical and inflammatory indexes before and after TEI medication; and 7) TEI-related adverse reactions, hospitalization time, and 28-day mortality.

### Efficacy Evaluation

According to the guiding principles of Clinical Trial Technology of antibacterial agents (2014 edition) (Writing Group of Guidance for Clinical Trials of Antibacterial Drugs, 2014), the judgment on efficacy evaluation includes clinical efficacy and microbiological efficacy. Clinical efficacy was evaluated after 7–14 days of medication. Clinical efficacy was evaluated in terms of clinical cure and clinical failure. Clinical cure is defined as the complete resolution or significant improvement of pretreatment infection signs and symptoms such that no additional antimicrobial therapy is needed. Clinical failure is defined as no apparent response or an incomplete response that needs additional antibiotic therapy for infections. The microbiological efficacy was evaluated as eradication, presumed eradication, no eradication, and presumed no eradication. Microbiological success was defined as eradication or presumed eradication of Gram-positive bacteria (Writing Group of Guidance for Clinical Trials of Anti-bacterial Drugs, 2014). The primary outcomes were clinical cure and microbiological success rates at the end of treatment (EOT).

### Influence Factors of the Eradication of Gram-Positive Bacteria

Univariate and multivariate logistic regression analyses were used to select the covariables of the regression model. We established the regression model for predicting the factors influencing the eradication of Gram-positive bacteria. In univariate analysis, the χ^2^ test or the Fisher exact test was used to compare the categorical variables. Continuous variables were compared by using the Mann–Whitney *U* test. Covariates with *p* values <0.1 were included in the multivariate logistic regression analysis (backward procedure, based on the *p*-value of the predictor removed) (Hammitt et al., 2019). Multivariate logistic regression analysis was used to screen for independent risk factors. SPSS software automatically screened out the independent variables when the optimal balance was reached between the fitting degrees of the prediction model.

Independent risk factor variables were used to establish regression equations and calculate prediction probabilities. The receiver operating characteristic (ROC) curves of multivariate factors were drawn; the area under the curve (AUC), cutoff point, Youden index, sensitivity, and specificity were used to evaluate their predictive value for predicting the factors influencing the eradication of Gram-positive bacteria.

### CRRT Parameters

All CRRT patients had selected femoral venipuncture catheterization as the vascular access, and the catheter used was an ARROW double-lumen central venous catheter. The blood purification equipment used was the Prisma Flex apparatus (Gambro/Baxter, Lakewood, co) (Baxter, USA). All CRRT filters used were polymethyl methacrylate (PMMA) membrane blood filters (CH-1.0) (Toray Medical, Japan). The CRRT mode was CVVH, with a blood flow of 180–200 ml/min, replacement fluid volume of 2–3 L/h, ultrafiltration volume of 100–300 ml/h, pre-dilution of 60–90%, subsequent dilution of 10–40%, and ultrafiltration rate of 30–45%.

### Effect of CRRT on Clearance of TEI

The blood and ultrafiltrate samples were taken on Day 2 or Days 6–8 of TEI therapy. One hour (C_max_) and 24 h after the start of infusion (C_min_), additional pre- and post-hemofilter blood and 10 ml ultrafiltrate samples from the ultrafiltrate outlet of the hemofilter were taken. The ultrafiltrate concentrations were also determined by using HPLC. This assay has been validated for ultrafiltrate samples using a spiked ultrafiltrate (1.0–30.0 μg/ml). The lower limit of quantification in the ultrafiltrate was 0.1 μg/ml. The precision of the assay was <9% (coefficient of variation).

The calculation formulas of the CRRT ultrafiltration rate (UFR) (ml/kgh) and TEI filtration coefficient for the first 48 h of CRRT during TEI treatment, according to the literature reports, were as follows (Naorungroj et al., 2021):

$$\text{Plasma flow rate}\left({\text{Q}}_{\text{p}}\right)=\text{BFR}\times \left(1-\text{HCT}\right),$$ (6)

$$\text{Dilution ratio}\left(\text{A}\right)=\frac{{\text{Q}}_{\text{P}}}{{\text{Q}}_{\text{P}}+\frac{{\text{Q}}_{\text{pre}}}{60}},$$ (7)

$$\text{Ultrafiltration rate}=\text{A}\times \frac{{\text{Q}}_{\text{pre}}+{\text{Q}}_{\text{post}}+{\text{Q}}_{\text{uf}}}{\text{Wt}},$$ (8)

$$\text{Filtration coefficient}\left(\text{Sc}\right)=\frac{{\text{C}}_{\text{uf}}}{{\text{C}}_{\text{s}}},$$ (9)

where Q_p_ is the plasma flow rate (ml/min), BFR is the blood flow rate (ml/min), HCT is the hematocrit, Q_pre_ is the flow rate of pre-replacement fluid pump (ml/h), Q_post_ is the flow rate of post-replacement fluid pump (ml/h), Q_uf_ is the water removal (ml/h), Wt is the body weight (kg) before medication, S_c_ is the TEI filtration coefficient, C_uf_ is the TEI concentration in ultrafiltrate (mg/L), and C_s_ is the blood concentration of TEI at the same time (mg/L).

The PK parameters were calculated by using a non-compartmental model and Kinetica-2000 (InnaPhase Corporation, France). The area under the concentration–time curve in 24 h (AUC_0–24_) was computed using the log-linear method or the trapezoidal method. Since the replacement fluid was applied simultaneously in pre-dilution and post-dilution modes, the hemofilter clearance CL_HF_ was determined as follows (Bellmann et al., 2010):

$${\text{CL}}_{\text{HF}}={\text{CL}}_{\text{HFpre}-\text{dilution}}+{\text{CL}}_{\text{HFpost}-\text{dilution}},$$ (10)

$${\text{CL}}_{\text{HFpre}-\text{dilution}}=\text{UFR}\times \text{Sc}\times \frac{\text{BFR}}{\text{BFR}+\text{SuR}},$$ (11)

$${\text{CL}}_{\text{HFpost}-\text{dilution}}=\text{UFR}\times \text{Sc,}$$ (12)

$${\text{AUC}}_{24\text{h}}\left(\text{mg}/\text{L}\cdot \text{h}\right)=\frac{\text{dailydose}\left(\text{mg}\right)}{\text{CL}\left(\text{L}/\text{h}\right)},$$ (13)

where CL_HF pre-dilution_ is the hemofilter clearance obtained by using the pre-dilution mode, CL_HF post-dilution_ is the hemofilter clearance obtained by using the post-dilution mode, UFR is the ultrafiltration rate, CUF is the TEI concentration in the ultrafiltrate, CS is the TEI serum concentration, BFR is the blood flow rate through the hemofilter, and SuR is the flow rate of the substitution solution.

### Statistical Analysis

All statistical analyses were performed using the Statistical Package for Social Sciences, version 22 (IBM, USA), and GraphPad Prism version 6 (GraphPad company, USA). The categorical variables were expressed as case numbers (n) and proportions (%). Pearson’s chi-square test was used to compare the categorical variables. All the continuous variables were checked for normality using the Shapiro–Wilk test. Continuous variables in accordance with the normal distribution were summarized as χ ± SD; the independent sample t-test was used to analyze these continuous data. The continuous variables were summarized as medians and interquartile ranges; the Mann–Whitney *U* test was used to analyze continuous data when these data were not normally distributed. The linear regression analysis was used to compare the correlation between two continuous variables. Two-tailed *p* < 0.05 were considered statistically significant.

## Results

### Patient Characteristics

A total of 106 cases of renal dysfunction patients who received anti-infective treatment with TEI were enrolled in this study, including 40 cases in the CRRT group and 66 cases in the non-CRRT group. The APACHEⅡscore was higher in the CRRT group and had statistical differences when compared with the non-CRRT group (*p* = 0.017). There were no statistical differences in other variables between the two groups (*p* > 0.05), as shown in Table 1.

**TABLE 1:** Baseline characteristics and anti-infection treatments in two groups.

Characteristics	Non-CRRT group (*n* = 66)	CRRT group (*n* = 40)	Statistical value	*p*-value
No. (%) male/no. (%) female	40 (60.6)/26 (39.4)	25 (62.5)/15 (37.5)	0.038	0.846
Median age (IQR), yr	79.0 (72.0–86.0)	83.0 (65.5–89.0)	−0.406	0.684
Median body weight (IQR), kg^a^	55.0 (52.0–65.0)	60.0 (55.0–67.5)	−1.314	0.189
Median APACHE Ⅱscore^a^ (IQR)	19.0 (13.0, 25.0)	23.0 (21.0, 32.5)	−2.396	0.017
Median lactic acid (IQR), mmol/L^a^	1.4 (0.8, 2.7)	2.2 (1.2, 3.2)	−1.810	0.070
Mean albumin (SD), g/L^a^	31.1 ± 6.7	30.8 ± 6.5	−0.290	0.772
Median loading dose (IQR), mg/kg, q12 h × 3 doses	10.0 (7.6–10.9)	10.0 (8.6–10.9)	−0.406	0.684
Median maintenance dose (IQR), mg/kg, qd	7.3 (5.7–8.9)	6.8 (6.0–8.0)	−0.611	0.542
Median duration of TEI therapy (IQR), d	11.5 (7.0–15.0)	11.0 (7.0–14.5)	−0.767	0.443
Combined antibiotics, no. (%)				
Meropenem	8 (12.1)	7 (17.5)	0.593	0.441
Imipenem	7 (10.6)	6 (15.0)	0.447	0.504
Piperacillin/tazobactam	7 (10.6)	5 (12.5)	0.089	0.765
Cefperazone/sulbactam	2 (3.0)	4 (10.0)	—	0.196
Levofloxacin	2 (3.0)	1 (2.5)	—	1.000
Moxifloxacin	2 (3.0)	0 (0.0)	—	0.526
In-hospital deaths	17 (25.8)	16 (40.0)	2.356	0.125

a At the start of TEI administration; CRRT, continuous renal replacement treatment; IQR, interquartile range; APACHE II score:Acute Physiology and Chronic Health Evaluation II score.

### Comparison of TEI C_min_

**FIGURE 1:**
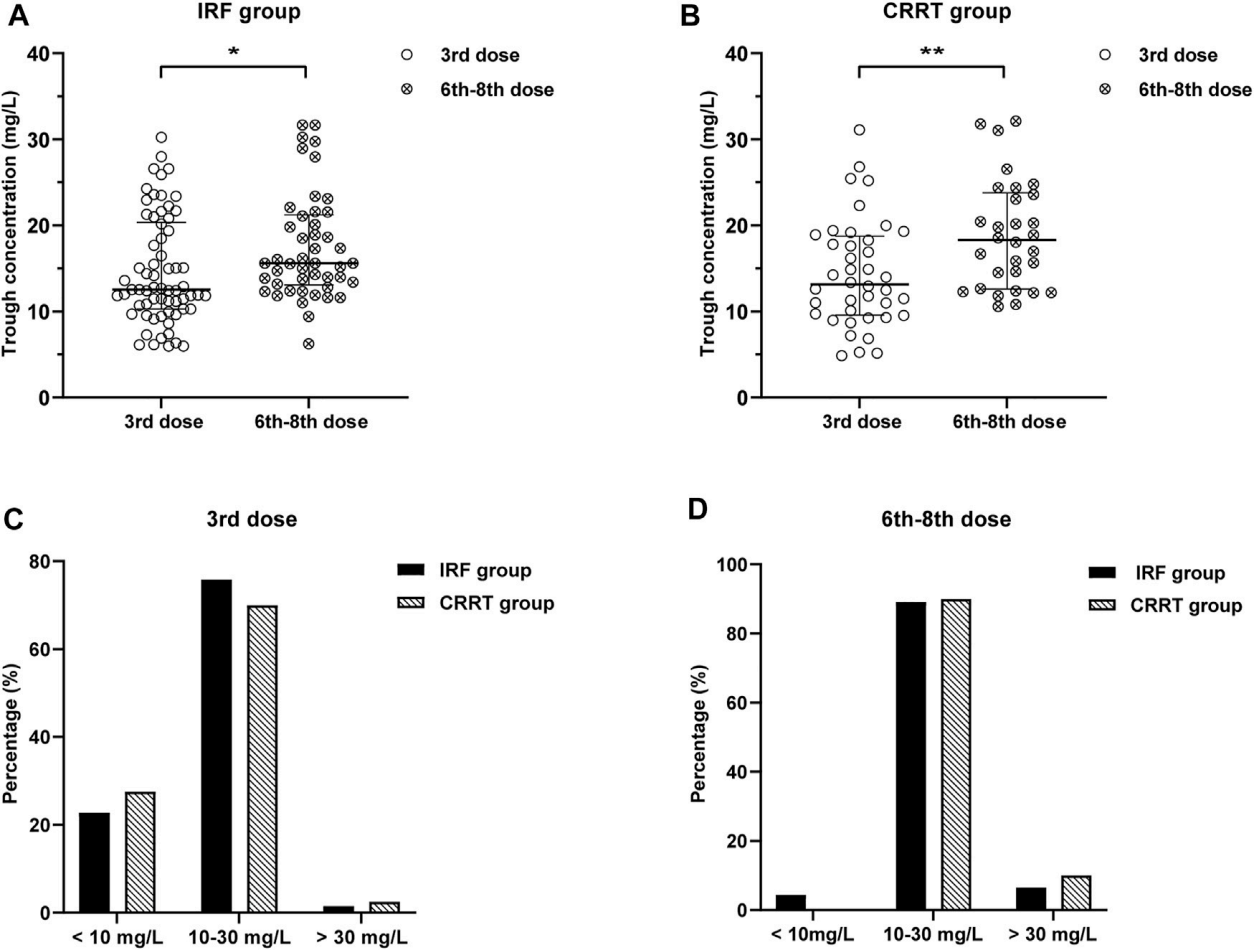
**(A,B)** Distribution of TEI C_min_ before the 3rd dose and the 6th–8th dose in the IRF and CRRT groups. C_min_ before the 6th–8th dose was much higher than that before the 3rd dose in the IRF and CRRT groups [16.2 (13.4–22.1) mg/L vs. 12.9 (10.7–20.8) mg/L, *p* = 0.028; 20.3 (10.8–24.8) mg/L vs. 12.5 (9.4–18.7) mg/L, *p* = 0.004]. **(C,D)** Rates of TEI C_min_ before the 3rd dose and the 6th–8th dose in the range of 10–30 mg/L were 75.8 and 89.1% in the IRF group, and 70.0 and 90.0% in the CRRT group, respectively.

There were 16 cases in the non-CRRT group that had the initial C_min_ out of the target range, and 11 cases had their dosage regimen of TEI adjusted. In the CRRT group, 12 cases had the initial C_min_ out of the target range, and 8 cases (86.9%) had the dosage regimen of TEI adjusted. A total of 76 trough concentrations were redetermined after adjusting the dosage regimen or the 6th–8th dose of the maintenance dosage regimen, 46 concentrations in the non-CRRT group, and 30 concentrations in the CRRT group. The rates of TEI C_min_ before the 3rd dose and the 6th–8th dose in the range of 10–30 mg/L were 75.8 and 89.1% in the IRF group, and 70.0 and 90.0% in the CRRT group, as shown in Figures 1C,D. The rate of C_min_ in the range of 10–30 mg/L was much higher before the 6th–8th dose than the 3rd dose in the CRRT group (90.0 vs. 70.0%, *p* = 0.044). The rates of C_min_ in the range of 15–30 mg/L before the 6th–8th dose were 71.7 and 70% in the non-CRRT and CRRT groups.

The distribution of TEI C_min_ before the 3rd and the 6th–8th dose in the non-CRRT and CRRT groups is shown in Figures 1A,B. C_min_ before the 6th–8th dose were much higher than that before the 3rd dose in the non-CRRT and CRRT groups [16.2 (13.4–22.1) mg/L vs. 12.9 (10.7–20.8) mg/L, *p* = 0.028; 20.3 (10.8–24.8) mg/L vs. 12.5 (9.4–18.7) mg/L, *p* = 0.004].

### Infection Sites and Distribution of Pathogens

The respiratory system was the most common site of infection in the two groups, followed by bloodstream and intra-abdominal infections, and there was no statistical difference in the distribution of infected sites between the two groups (Table 2). Blood and sputum were the main culture specimens of Gram-positive bacteria. All Gram-positive bacteria were resistant to penicillin and methicillin. *Enterococcus faecium* was the most common pathogen in the target treatment of the two groups, followed by methicillin-resistant coagulase-negative staphylococci (MRCNS) and MRSA. There were no statistical differences in pathogen distribution and MIC values between the two groups, as shown in Table 3.

**TABLE 2:** Site of infection and the distribution of Gram-positive bacteria.

Characteristics	Non-CRRT group (*n* = 66)	CRRT group (*n* = 40)	Statistical value	*p*-value
Infection site, no. (%)				
Respiratory	52 (78.8)	34 (85.0)	0.628	0.428
Blood stream	26 (39.4)	14 (35.0)	0.205	0.651
Sepsis shock	21 (31.8)	18 (45.0)	1.861	0.173
Intra-abdominal	17 (25.8)	14 (35.0)	1.028	0.311
Urinary system	11 (16.7)	9 (22.5)	0.554	0.457
Skin and soft tissue	7 (10.6)	5 (12.5)	0.089	0.765
Infective endocarditis	3 (4.5)	2 (5.0)	—	1.000
More than two infection sites	30 (45.5)	21 (52.5)	0.495	0.482
The specimen source of G^+^, no. (%)				
Blood	21 (30.9)	16 (35.6)	0.269	0.604
Sputum	21 (30.9)	12 (26.7)	0.233	0.629
Drainage fluid	9 (13.2)	6 (13.3)	0.000	0.988
Urine	11 (16.2)	6 (13.3)	0.229	0.632
Wound secretion	6 (8.8)	5 (11.1)	0.161	0.688
Distribution of bacteria, n (%)				
*Enterococcus*	29 (42.6)	16 (35.6)	0.568	0.451
MRCNS	22 (32.4)	16 (35.6)	0.124	0.724
MRSA	17 (25.0)	13 (28.9)	0.210	0.647
Mixed with other pathogens	19 (28.8)	13 (32.5)	0.163	0.687
Distribution of MIC, n (%)				
≤1 mg/L	65 (95.6)	42 (93.3)	—	0.681
2 mg/L	2 (2.9)	2 (4.4)	—	1.000
4 mg/L	1 (1.5)	1 (2.2)	—	1.000

CRRT, continuous renal replacement treatment; MRCNS, methicillin-resistant coagulase-negative staphylococci; MRSA, methicillin-resistant Staphylococcus aureus; MIC, minimum inhibitory concentration.

**TABLE 3:** Univariate regression analysis of risk factors on clearance of Gram-positive bacteria.

Variables	G^+^ uncleared (*n* = 21)	G^+^ cleared (*n* = 85)	*p*-value	OR	95% CI
No. (%) male/no. (%) female	12 (57.1)/9 (42.9)	53 (62.4)/32 (37.6)	0.661	1.242	0.471–3.274
Median age (IQR), yr	82.5 (75.2–85.2)	78.0 (70.0–88.0)	0.319	0.979	0.938–1.021
Median body weight (IQR), kg^a^	58.0 (53.8–60.0)	60.0 (55.0–68.5)	0.256	1.031	0.978–1.087
Median APACHE Ⅱscore ^a^ (IQR)	32.0 (27.5, 36.0)	22.0 (17.0, 28.0)	0.015	1.131	1.024–1.250
Median lactic acid (IQR), mmol/L^a^	2.2 (1.4, 3.4)	1.4 (0.9–2.9)	0.439	1.138	0.821–1.577
Mean of the 1st albumin (SD), g/L^a^	28.9 ± 5.6	31.2 ± 6.6	0.010	1.158	1.036–1.295
Mean of the 2nd albumin (SD), g/L^b^	29.8 ± 4.6	33.8 ± 5.3	0.001	1.273	1.106–1.467
Median TBIL (IQR), umol/L	14.0 (7.2–34.6)	10.2 (6.2–21.5)	0.494	0.994	0.978–1.011
Median ALT (IQR), U/L	28.0 (19.0–68.5)	37.5 (23.0–67.8)	0.624	0.999	0.997–1.002
Median baseline of Cr (IQR), umol/L	126.0 (86.0–163.0)	113.0 (84.8–162.0)	0.844	0.999	0.994–1.005
Median Cr at withdrawal (IQR), umol/L	90.3 (66.0, 127.5)	93.0 (70.0, 133.5)	0.331	0.996	0.988–1.004
Median duration of TEI therapy (IQR), d	7.5 (7.0–10.2)	9.0 (7.0–13.0)	0.021	1.228	1.032–1.460
Median of the 1st concentration (IQR), mg/L^c^	10.3 (9.1–12.6)	13.6 (11.1–20.5)	0.005	1.176	1.050–1.317
Median of the 2nd concentration (IQR), mg/L^b^	14.4 (12.4–18.4)	17.3 (12.7–23.2)	0.024	1.138	1.017–1.273
Infection site and complication, n (%)					
Respiratory	16 (76.2)	70 (82.4)	0.591	1.367	0.437–4.282
Blood stream	8 (38.1)	32 (37.6)	0.940	1.038	0.388–2.776
Sepsis shock	6 (28.6)	33 (38.8)	0.383	1.100	0.423–2.859
Intra-abdominal	5 (23.8)	26 (30.6)	0.641	0.769	0.255–2.317
Urinary system	3 (14.3)	17 (20.0)	0.651	0.735	0.194–2.782
Skin and soft tissue	3 (14.3)	9 (10.6)	0.548	1.537	0.378–6.249
Infective endocarditis	1 (4.8)	4 (4.7)	0.926	1.112	0.118–10.496
Mechanical ventilation	13 (61.9)	47 (55.3)	0.348	1.59	0.603–4.196
MODS	10 (47.6)	14 (16.5)	0.002	0.195	0.070–0.545
More than two infection sites	7 (33.3)	44 (51.8)	0.248	0.557	0.206–1.505
The specimen source of G^+^ - n (%)	23	90			
Blood	7 (30.4)	30 (33.3)	0.893	0.933	0.340–2.562
Sputum	8 (34.8)	25 (27.8)	0.388	1.551	0.573–4.195
Drainage fluid	2 (8.7)	13 (14.4)	0.566	0.632	0.131–3.038
Urine	3 (13.0)	14 (15.6)	0.899	0.917	0.238–3.530
Wound secretion	3 (13.0)	8 (8.9)	0.450	1.729	0.417–7.164
Distribution of bacteria, n (%)					
*Enterococcus*	10 (43.5)	35 (38.9)	0.420	1.481	0.570–3.844
MRCNS	8 (34.8)	30 (33.3)	0.587	1.313	0.492–3.504
MRSA	5 (21.7)	25 (27.8)	0.793	0.863	0.286–2.600
Mixed with other pathogens	6 (26.1)	26 (28.9)	0.933	1.046	0.366–2.987
Distribution of MIC, n (%)					
≤1 mg/L	21 (91.3)	86 (95.6)	0.481	0.540	0.097–2.994
2 mg/L	1 (4.3)	3 (3.3)	0.724	1.517	0.150–15.346
4 mg/L	1 (4.3)	1 (1.1)	0.284	4.650	0.279–77.514
Combined with antibiotics, n (%)	10 (47.6)	41 (48.2)	0.650	1.290	0.430–3.870
Mean loading dose of TEI (SD), mg/kg, q12 h × 3 doses	9.5 ± 1.9	9.0 ± 2.3	0.373	0.901	0.717–1.133
Mean maintenance dose of TEI (SD), mg/kg, qd	7.1 ± 2.2	7.3 ± 2.1	0.640	1.056	0.839–1.329

a Before the start of TEI administration.

b Before the 6th–8th dose of TEI administration.

c Before the 3rd dose of TEI administration; OR, odds ratio; 95% CI, 95% confidence interval; APACHE II score:Acute Physiology and Chronic Health Evaluation II score; TBIL, total bilirubin; ALT, alanine transaminase; Cr, creatinine; MODS, multiple organ dysfunction syndrome; MRCNS, methicillin-resistant coagulase-negative staphylococci; MRSA, methicillin-resistant Staphylococcus aureus; MIC, minimum inhibitory concentration.

### Clinical Outcomes in Patients Cured of Gram-Positive Bacteria Infection

Clinical outcome indicators are shown in Table 3. After TEI treatment, 46 cases were clinically cured in the non-CRRT group, 27 cases were clinically cured in the CRRT group, and there were no statistical differences (72.7 vs. 67.5%, *p* = 0.566). The eradication rates of Gram-positive bacteria were 81.8% (48 cases confirmed eradication and 6 cases presumed) in the non-CRRT group, 77.5% (27 cases confirmed eradication and 4 cases presumed) in the CRRT group, and had no statistical differences (81.8 vs. 77.5%, *p* = 0.589). Gram-positive bacteria were not eradicated in 21 cases totally. There were 4 cases that developed acute kidney injury (AKI) and one case of liver injury in the non-CRRT group after TEI treatment. Two cases of liver injury occurred in the CRRT group, and there were no statistical differences in the rate of adverse events (7.6 vs. 5.0%, *p* = 0.909). The 28-day hospital mortality rate also had no statistical significance in the CRRT and non-CRRT groups (22.5 vs. 15.2%, *p* = 0.339).

### Influence Factors of the Eradication of Gram-Positive Bacteria

The total 106 cases were divided into G^+^ uneradicated (21 cases) and G^+^ eradicated groups (85 cases) according to whether the Gram-positive bacteria were eradicated. The univariate analysis indicated that the APACHEⅡscore, lower albumin levels before the start of TEI administration and before the 6th–8th dose, lower C_min_ before the 3rd dose and the 6th–8th dose, the shorter duration of TEI therapy, and more of MODS were related with uneradicated G^+^ bacteria, and had statistically significant differences (*p* < 0.05) (Table 3).

The risk factors for the G^+^ uneradicated group were estimated by the step-wise multivariate linear regression analysis (backward procedure, based on the *p*-value of the predictor removed) and the identification of the cutoff values with ROC curve analyses. Variables with *p* < 0.1 in the univariate results were estimated in the multivariate analysis. The significant independent factors for the G^+^ uneradicated group were the APACHEⅡscore, lower albumin levels before TEI treatment, lower C_min_ before the 3rd dose, and more of MODS (*p* < 0.05) (Table 4). After the introduction and elimination of the above independent variables, the logistic regression equation was finally established:

$$\text{Logit(p)}=0.220\times \text{APACHE}Ⅱ\text{score}+2.292\times \text{MODS}-0.223\times \text{ALB}-0.122\times {\text{C}}_{\text{min}}+9.6,$$ (14)

**TABLE 4:** Results of risk factors on clearance of Gram-positive bacteria screened by multivariate logistic regression analysis.

Variables	*p*-value	OR	95% CI
APACHE Ⅱscore^a^	0.027	1.428	1.040–1.959
The 1st albumin (g/L)^a^	0.032	0.726	0.542–0.972
The 2nd albumin (g/L)^b^	0.883	0.971	0.655–1.439
Duration of TEI therapy (d)	0.465	0.861	0.575–1.288
The 1st concentration (mg/L)^c^	0.035	0.524	0.287–0.956
The 2nd concentration (mg/L)^b^	0.535	1.170	0.713–1.920
MODS	0.030	11.065	2.743–46.927

a Before the start of TEI administration.

b Before the 6th–8th dose of TEI administration.

c Before the 3rd dose of TEI administration; OR, Odds ratio; 95% CI, 95% confidence interval; APACHE II score:Acute Physiology and Chronic Health Evaluation II score; MODS, multiple organ dysfunction syndrome.

**FIGURE 2:**
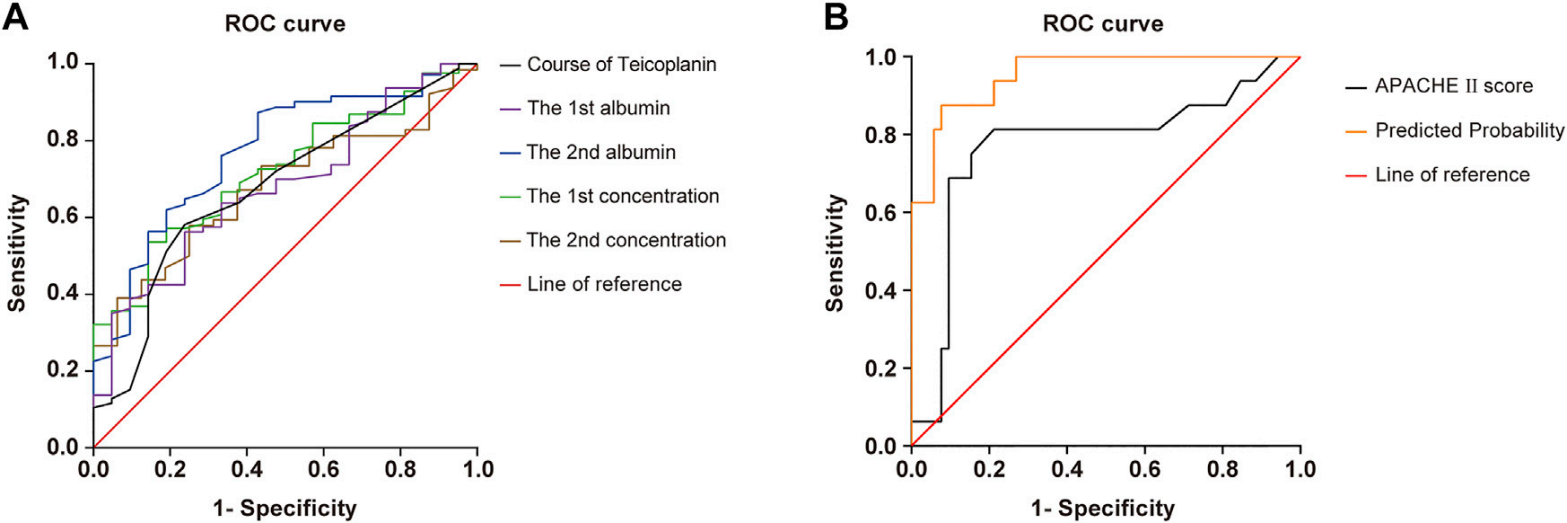
ROC curve of predicted probability, APACHE Ⅱscore at admission, course of TEI, the 1st albumin, the 2nd albumin, the 1st concentration, and the 2nd concentration, which were independent risk factors, and combined predictor on clearance of Gram-positive bacteria. The predicted probability of the APACHE Ⅱscore (AUC = 0.772), the course of TEI (AUC = 0.646), the 1st albumin (AUC = 0.758), the 2nd albumin (AUC = 0.764), the 1st concentration (AUC = 0.710), and the 2nd concentration (AUC = 0.666) had better sensitivity (87.5%) and specificity (92.3%), Youden index of 0.798, as well as the maximal AUC (AUC = 0.954).

For each independent risk factor of continuous variables, we calculated the specificity and sensitivity of the resulting logistic regression model by constructing ROC curves and calculating the AUC statistic to estimate the model’s ability to identify uneradicated G^+^ bacteria. The ROC curves of the APACHEⅡscore, the albumin level, C_min_, the course of TEI treatment, and the predicted probability were drawn (Figure 2). The area under the ROC curve, cutoff point, Youden index, sensitivity, specificity, and predicted probability are shown in Table 5. An APACHEⅡscore of more than 22.5, the albumin levels before the start of TEI administration and before the 6th–8th dose lower than 32.8 g/L and 29.3 g/L, C_min_ before the 3rd dose and the 6th–8th dose lower than 13.2 mg/L and 17.1 mg/L, and the duration of TEI therapy being shorter than 10.5 days had more risk of uneradicated G^+^ bacteria. The area under the ROC curve of the predicted probability was 0.954, which was higher than the other six factors, the Youden index was 0.798, and the sensitivity and specificity were 87.5 and 92.3%, respectively.

**TABLE 5:** Area under the curve and cutoff values of receiver operating characteristic curve for predicting effects on clearance of Gram-positive bacteria.

Variables	AUC (95%CI)	*p*-value	Cutoff point	Youden index	Sensitivity (%)	Specificity (%)
APACHE Ⅱscore^a^	0.772 (0.619–0.924)	0.001	22.5	0.601	81.3	78.8
The 1st albumin (g/L)^a^	0.758 (0.641–0.875)	0.002	32.8	0.481	48.1	100
The 2nd albumin (g/L)^b^	0.764 (0.633–0.896)	0.001	29.3	0.534	84.6	68.8
Duration of TEI therapy (d)	0.646 (0.497–0.795)	0.079	10.5	0.332	51.9	81.3
The 1st concentration (mg/L)^c^	0.710 (0.584–0.835)	0.012	13.2	0.413	53.8	87.5
The 2nd concentration (mg/L)^b^	0.666 (0.532–0.800)	0.046	17.1	0.327	57.7	75
Predicted probability	0.954 (0.906–1.000)	0.000	0.245	0.798	87.5	92.3

a Before the start of TEI administration.

b Before the 6th–8th dose of TEI administration.

c Before the 3rd dose of TEI administration; APACHE II score:Acute Physiology and Chronic Health Evaluation II score; AUC: area under the curve.

### Relationship Between Albumin Levels and TEI C_min_

**FIGURE 3:**
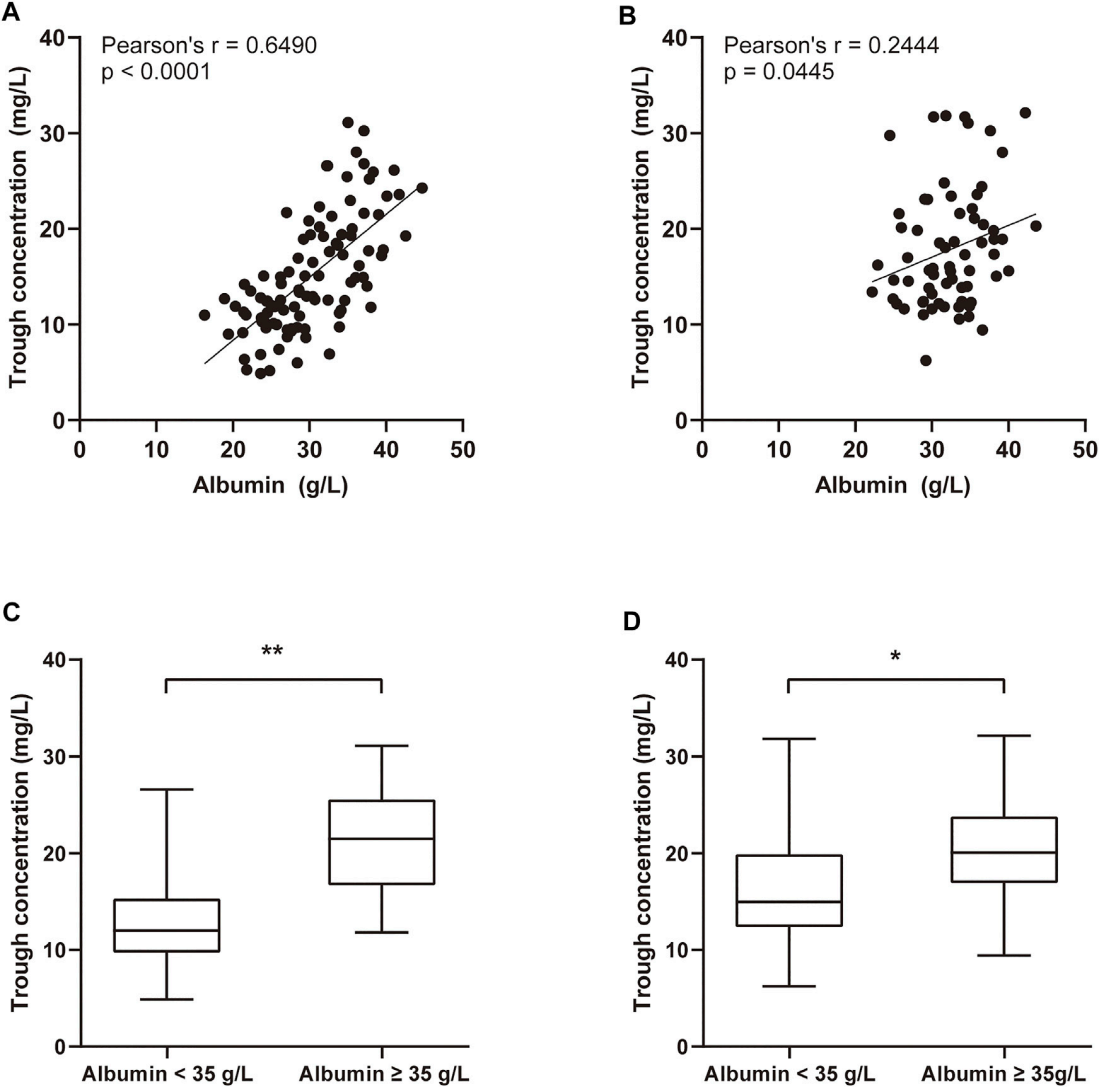
The linear regression analysis was used to compare the correlation between the albumin level and C_min_ of TEI. **(A)** The correlation coefficient (r) was 0.6490 between the C_min_ before the 3rd dose and the albumin level before the start of TEI administration (*p* < 0.001). **(B)** The correlation coefficient (r) was 0.2444 between the C_min_ before the 6th–8th dose and the albumin level before the 6th–8th dose (*p* = 0.0445). **(C)** The C_min_ before the 3rd dose was much lower in the albumin <35 g/L group than in the albumin ≥35 g/L group [12.0 (9.7–15.3) vs. 21.5 (16.7–25.6), *p* = 0.000]. **(D)** The C_min_ before the 6th–8th dose was also lower in the albumin <35 g/L group than in the albumin ≥35 g/L group [15.0 (12.3–19.9) vs. 20.0 (16.9–23.8), *p* = 0.011].

The linear regression analysis was used to assess the correlation between the albumin level and C_min_ of TEI. The correlation coefficient (r) was 0.6490 between C_min_ before the 3rd dose and the albumin level before the TEI administration (*p* < 0.001). The correlation of the albumin level with C_min_ before the 3rd dose was much better than that of the albumin level with C_min_ before the 6th–8th dose (Figures 3A,B). The C_min_ before the 3rd dose in the albumin <35 g/L group was much lower than that in the albumin ≥35 g/L group [12.0 (9.7–15.3) vs. 21.5 (16.7–25.6), *p* = 0.000]. The C_min_ before the 6th–8th dose in the albumin <35 g/L group was also lower than that in the albumin ≥35 g/L group [15.0 (12.3–19.9) vs. 20.0 (16.9–23.8), *p* = 0.011].

### The Effect of CRRT on Clearance of TEI

**FIGURE 4:**
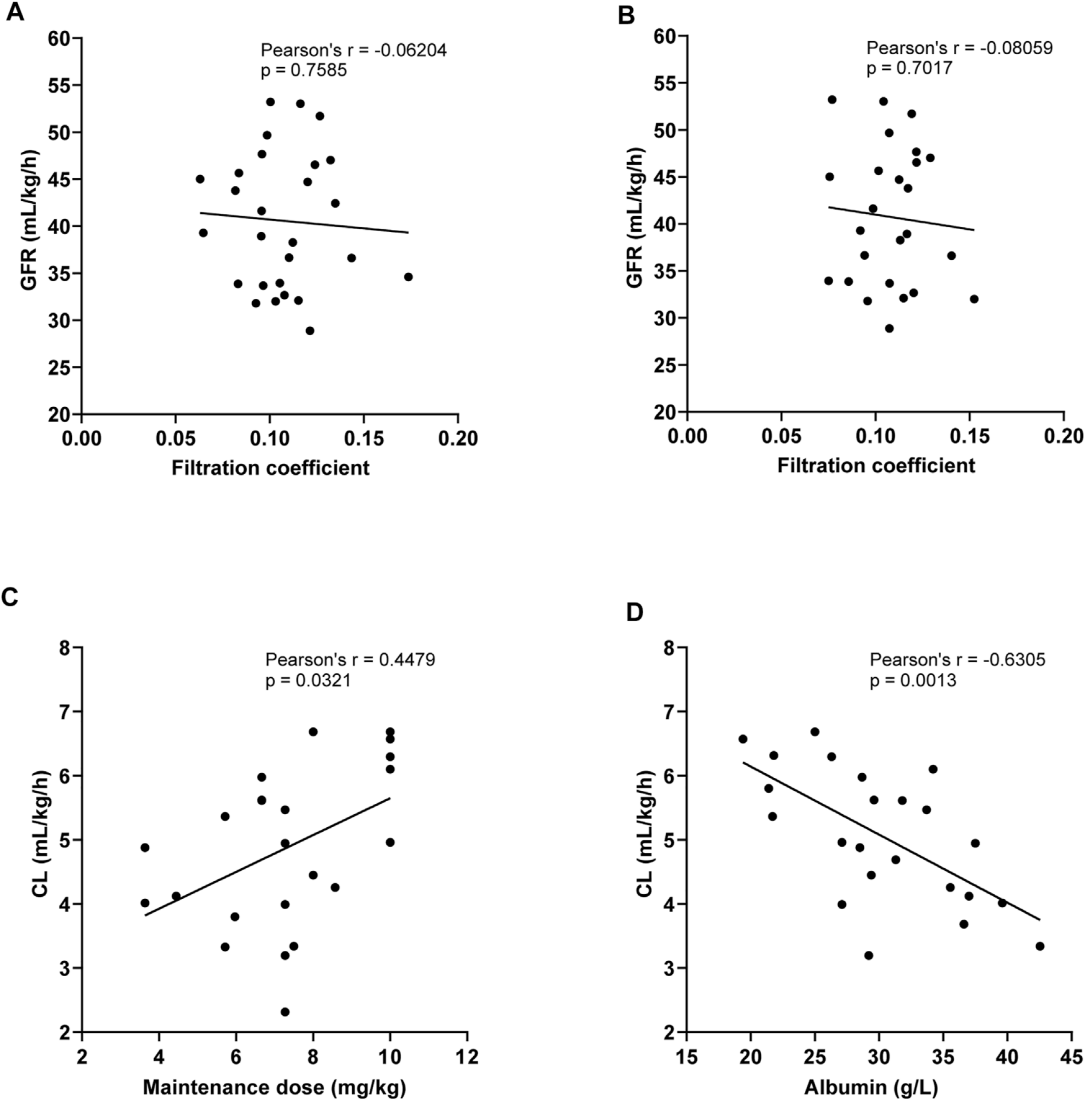
**(A,B)** Linear regression analysis was used to compare the correlation between the GFR and filtration coefficient at the 6th–8th dose of TEI C_min_ **(A)** or C_max_ **(B)**. The GFR had no correlation with the filtration coefficient (r = −0.06204, *p* = 0.7585; r = −0.08059, *p* = 0.7017). **(C)** The clearance of TEI in CRRT patients was positively correlated with the maintenance dose of TEI (r = −0.4479, *p* = 0.0321). **(D)** The clearance of TEI in CRRT patients was negatively correlated with the albumin level (r = −0.6305, *p* = 0.0013).

There were 26 patients in the CRRT (CVVH) group who had their C_max_, C_min_, and filtrate concentrations determined at the same time, as shown in Table 6. The ultrafiltration rate was 44.7 ± 10.1 ml/kgh. The filtration coefficient at the peak concentration was 11.1 ± 2.5 mg/L, and it had no statistical differences when compared with that at C_min_ (*p* = 0.560). The linear regression analysis was used to compare the correlation between GFR and filtration coefficient at the 6th–8th dose of TEI C_min_ or C_max_. It was shown that the GFR had no correlation with the filtration coefficient (r = −0.06204, *p* = 0.7585; r = −0.08059, *p* = 0.7017) (Figures 4A,B). However, the clearance of TEI in CRRT patients was positively correlated with the maintenance dose of TEI (r = −0.4479, *p* = 0.0321) and negatively correlated with the albumin level (r = −0.6305, *p* = 0.0013) (Figures 4C,D).

**TABLE 6:** CRRT parameters and TEI concentration in CRRT patients.

	CRRT patients (n = 26)
Mean CRRT parameters (SD)	
Hematocrit (%)	25.2 ± 4.1
Blood flow rate (ml/min)	171.5 ± 18.3
Pre-dilution flow rate (ml/h)	1925.6 ± 357.5
Post-dilution flow rate (ml/h)	991.0 ± 208.6
Negative equilibrium per hour (ml/h)	187.0 ± 63.7
Ultrafiltration rate (ml/kg/h)	44.7 ± 10.1
Mean concentration of TEI (SD), mg/L	
Peak serum concentration at the 6th–8th dose	35.3 ± 7.2
Peak filtrate concentration at the 6th–8th dose	3.9 ± 1.1
Filtration coefficient at peak concentration (%)	11.1 ± 2.5^a^
Trough serum concentration at the 6th–8th dose	23.8 ± 7.1
Trough filtrate concentration at the 6th–8th dose	2.5 ± 0.8
Filtration coefficient at C_min_ (%)	10.7 ± 2.4
Mean pharmacokinetic parameter of TEI (SD), mg/L	
CL (ml/h/kg)	4.9 ± 1.3
AUC (ug·h/ml)	1541.8 ± 507.4
AUC/MIC (MIC = 2)	770.9 ± 253.7

a Compared with filtration coefficient at C_min_; t = 0.586, *p* = 0.560; CL, clearance; AUC, area under the curve; MIC, minimum inhibitory concentration.

We also evaluated the correlation of the lactic acid level and APACHE Ⅱscore with C_min_ of TEI; it showed that the lactic acid level and APACHE Ⅱ score had no correlation with C_min_ of TEI (r = 0.051, *p* = 0.690; r = 0.034, *p* = 0.843).

## Discussion

Some studies suggested that patients with renal dysfunction need a high-loading dose of TEI to achieve a target C_min_ in early stages of treatment (Nakamura et al., 2015; Ueda et al., 2016). Ueda et al. (2012) reported that only 20.3% of patients with renal dysfunction achieve the target C_min_ of 15 μg/ml by employing the conventional TEI-loading regimen. In the next study, Ueda T proposed a high-loading dose (10 mg/kg, q12 h × 2 doses) regimen in patients with renal dysfunction. These results indicate that employing target therapeutic PK is necessary for improved clinical efficacy (Ueda et al., 2016). However, even a higher loading dose regimen (12 mg/kg, q12 h × 4 doses) of TEI in patients with renal dysfunction resulted in a greater rate of C_min_ (more than 30 mg/L) and higher incidence of adverse effects (Nakamura et al., 2015). In our study, a high-loading dose (8–10 mg/kg, q12 h × 3 doses) of TEI achieved C_min_ within 24 h of the first administration to 10–30 mg/L in more than 70% of the patients, indicating that the therapeutic concentration of TEI can be reached quickly with this loading dosage regimen.

Also, more than 70% of the C_min_ reached 15–30 mg/L before the 6th–8th dose in our study. The C_min_ before the 6th–8th dose was much higher than that before the 3rd dose, indicating that TEI was accumulated in renal dysfunction patients, which needed reducing the maintenance dose. In two studies, the maintenance dose of TEI was recommended to be adjusted individually according to the TDM results of renal dysfunction patients (Nakamura et al., 2015; Ueda et al., 2016). For patients with CVVHDF and the high-flux CVVH mode of CRRT partly eliminating TEI with high variability, the optimized maintenance dose was not recommended for adult patients with renal dysfunction and CRRT (Bellmann et al., 2010; Lim et al., 2019; Oda et al., 2020). In our study, the initial maintenance dose of CRRT (CVVH mode) was designed according to CrCl in 30–60 ml/min/1.73 m^2^, and the TDM results indicated the maintenance dosage regimen was appropriate.

Some studies indicated that the albumin level, body weight, and renal function have significant effects on free TEI plasma concentrations and PK parameters (Yano et al., 2007; Brink et al., 2015; Emoto et al., 2021). Hypoalbuminemia significantly reduced concentrations of free TEI; however, it had no effect on total TEI concentrations (Brink et al., 2015). In our study, the albumin level was significantly correlated with C_min_ of total TEI, especially better with the initial C_min_. The C_min_ was much lower in the albumin <35 g/L group than in the albumin ≥35 g/L group. These results indicated that increasing the level of albumin can significantly increase the initial C_min_ of TEI.

C_min_ of TEI had a significant correlation with clinical efficacy. Initial C_min_ ≥ 15 μg/ml was an independent factor for increasing clinical efficacy. Achieving C_min_ ≥ 15 mg/L with dosage adjustment after TDM in patients with initial C_min_ <15 mg/L had no significant improvement in clinical efficacy (Ueda et al., 2016). Kim et al. (2019) performed a retrospective study of TEI TDM, focusing on the dose–serum concentration relationship and clinical outcomes, indicating that a high dose regimen and high mean C_min_ over 10 days were significantly associated with treatment efficacy. However, higher C_min_ was associated with adverse events during treatment. Routine TDM can be helpful in optimizing TEI administration. However, the reports about other factors affecting clinical efficacy were limited. In our study, the APACHE Ⅱscore, the albumin level, C_min_, the duration of TEI therapy, and MODS had effects on the eradication of Gram-positive bacteria. The APACHE Ⅱscore was designed as a mortality prediction tool, and for classifying patients by disease severity, some studies indicated that the APACHE Ⅱscore was significantly correlated with the efficacy of tigecycline (Fan et al., 2020), similar to our study. The albumin level and C_min_ at the early stage of TEI treatment had better correlation with the clearance of Gram-positive bacteria than that before the 6th–8th dose. These results indicated that a high level of C_min_ and albumin at the early stage of TEI treatment could significantly improve clinical efficacy. The regression model had a high prediction value for predicting clinical efficacy.

TEI elimination by CRRT depends on the technique and the protocol applied. Lim et al. (2019) assessed the effect of CVVHDF on the pharmacokinetics of TEI as maintenance therapy. The results showed that CL of TEI in the CVVHDF mode was 5.8 ± 4.2 ml/h/kg, which was higher than that of CVVH and the CVVHD mode (Bellmann et al., 2010). The size of the unbound TEI fraction and trough concentrations correlates inversely with the plasma albumin level (Yano et al., 2007; Brink et al., 2015; Ueda et al., 2020). However, no studies have confirmed whether hypoalbuminemia enhanced hemofilter clearance. In our study, the CL of TEI in the CVVH mode was 4.9 ± 1.3 ml/h/kg, which was lower than the literature reportings on the CVVHDF mode (Bellmann et al., 2010). The CL of TEI in the CVVH mode was positively correlated with the maintenance dose of TEI and negatively correlated with the albumin level. These results indicated that hypoalbuminemia also enhances the clearance of TEI in CRRT patients. The filtration coefficient of TEI was about 10% or higher, indicating that the molecular weight of TEI is large, and the rate of clearance by CRRT is limited. CRRT can promote the elimination of toxins (serum creatinine, lactic acid, and urea nitrogen) from the body (Wang et al., 2021), which partly explains the reason for no correlation between lactic acid and C_min_ of TEI.

Our study has several limitations that need to be considered. First, the number of patients was small, although previous studies on TEI in CRRT had even smaller numbers of patients. Second, urine TEI concentration was not measured, and the effect of residual renal function on TEI clearance was not adequately assessed. Third, we missed C_max_ and C_min_ for 14 cases of CRRT patients due to the following conditions: CRRT was stopped, changed the CVVH to another mode, and switched to IRRT.

## Conclusion

The therapeutic concentration range of TEI can be reached quickly with a high-loading dosage regimen in renal dysfunction and CRRT patients. The albumin level in the early stages can significantly affect the initial C_min_, clinical efficacy of TEI, and the clearance of TEI by CRRT. The amelioration of hypoproteinemia could reduce the variation of TEI PK/PD parameters. The filtration coefficient of TEI was stable at about 10%, even at a higher ultrafiltration rate. The loading dose and individualized maintenance dosage regimen in our study are appropriate and can be used as a reference to TEI treatment in renal dysfunction and CRRT (CVVH mode) patients.

## Data Availability

The raw data supporting the conclusion of this article will be made available by the authors, without undue reservation.
